# AI-driven vibration-based event classification in railway switches and crossings

**DOI:** 10.1038/s41598-026-58967-0

**Published:** 2026-06-23

**Authors:** Mohammad Adoul Amin, Taoufik Najeh, Abdelhamid Ghoul

**Affiliations:** 1 Division of Operation and Maintenance Engineering, Luleå University of Technology, Luleå, 971 87 Sweden; 2 Division of Signals and Systems, Luleå University of Technology, Luleå, 971 87 Sweden

**Keywords:** Railway switches and crossings, Vibration-based classification, Machine learning benchmarking, Autoencoder augmentation, Condition monitoring, Engineering, Mathematics and computing

## Abstract

Automated condition monitoring of railway switches and crossings (S&C) requires classification models whose reported accuracy reflects genuine generalization rather than evaluation artefacts. This paper presents a methodologically rigorous, leak-free machine-learning framework for vibration-based event classification, evaluated on accelerometer data from a full-scale outdoor S&C test facility. The pipeline enforces strict ordering (split, select, augment, standardize, train, evaluate) and partitions the data at the level of physical events, so that all measurements of a given event are assigned together to either the training or the test subset. A symmetric tabular autoencoder generates synthetic minority-class samples through latent-space interpolation. Twenty-one classifiers spanning eight families are benchmarked on held-out data and by group-aware five-fold cross-validation. The strongest models reach 81.5% held-out accuracy (ROC-AUC $\approx 0.94$) and $80.4\%\pm 2.1\%$ under cross-validation; ensemble methods are the most stable. Feature standardization is essential: without it, neural networks collapse below chance level. Computational profiling (inference latency 0.005–0.63 ms per one-second segment; model size 0.002–2.4 MB) maps three deployment scenarios to specific algorithm recommendations. Because the minority crossing class has only six held-out samples, its per-class metrics carry wide confidence intervals and should be interpreted with caution.

## Introduction

Railway switches and crossings (S&C) are among the most critical and failure-prone components of railway infrastructure, accounting for a disproportionate share of service disruptions and maintenance costs^1,2^. As network utilization intensifies globally, automated condition monitoring systems capable of classifying infrastructure events (crossings, rail joints, and squats) have become essential for transitioning from time-based to condition-based maintenance strategies^3–5^. Vibration-based monitoring, leveraging accelerometers mounted on or near the track, offers a cost-effective and non-intrusive modality for capturing the dynamic signatures associated with different fault types^6,7^, though sensor deployment in harsh railway environments presents significant challenges related to electromagnetic interference, temperature extremes, and data quality assurance^8–10^.

Machine learning (ML) approaches for vibration-based fault classification have advanced rapidly. Unsupervised clustering methods have been applied to wheel condition monitoring^11^ and track geometry anomaly detection^12^, while IoT-based sensor systems have enabled autonomous fault detection and localization^13^. Deep neural networks and audio-video fusion approaches have also demonstrated promise for railway defect identification^14^. In our earlier work^15,16^, we demonstrated the feasibility of ML-based event detection in railway infrastructure using multi-sensor vibration data, establishing baseline classification performance that motivates the comprehensive benchmarking presented here. Recent reviews by Chen et al.^17^ and Tang et al.^4^ highlight the breadth of data-driven methods applied to railway health monitoring. However, several critical methodological gaps persist.

First, many studies apply data-dependent preprocessing steps (feature selection, data augmentation, normalization) to the entire dataset before partitioning into training and test subsets. This introduces data leakage, whereby information from the test set influences model training, producing optimistically biased performance estimates that do not reflect genuine generalization capability^18^. The consequences are concrete: in safety-critical railway monitoring, an inflated accuracy estimate can lead an operator to set defect-detection thresholds too loosely, so that genuine defects are missed in service while the system is believed to be performing well. Kapoor and Narayanan^18^ document how leakage has produced irreproducible results across many ML-based scientific studies. In our preliminary experiments, correcting leakage altered accuracy estimates by several percentage points for some model families.

Second, railway fault datasets are inherently small and class-imbalanced. Fault events (crossings, joints, squats) occur far less frequently than normal-operation passages, and the cost of acquiring labeled data from full-scale test facilities is substantial. Data augmentation via generative models, including variational autoencoders and GANs, has been proposed to address this imbalance^19^, but the interaction between augmentation strategy and downstream classifier performance has not been systematically evaluated across multiple algorithm families.

Third, while individual studies often report results for a small number of classifiers, comprehensive multi-algorithm benchmarks that span tree-based, boosting, linear, instance-based, neural network, probabilistic, SVM, and meta-ensemble families are rare. Such benchmarks are essential for identifying the algorithm family best suited to the specific characteristics of railway vibration features. Moreover, computational profiling (training time, inference latency, memory footprint, model size) is seldom reported alongside accuracy metrics, despite being crucial for deployment decisions in resource-constrained edge computing environments^20,21^.

Fourth, the role of feature standardization is frequently overlooked. Railway vibration features span different numerical ranges; without normalization, scale-sensitive algorithms (neural networks, SVMs, KNN, linear classifiers) may fail to converge. Tree-based methods, which are invariant to monotonic feature transformations, mask this issue when used exclusively.

To address these gaps, this study presents a comprehensive ML framework for vibration-based event classification in railway S&C. The primary contributions are:

1. A leak-free evaluation pipeline that enforces the ordering split $\rightarrow$ select $\rightarrow$ augment $\rightarrow$ standardize $\rightarrow$ train $\rightarrow$ evaluate, and that partitions the data at the level of physical events so that no event is split between the training and test subsets.
2. A class-balanced autoencoder augmentation strategy that addresses minority-class under-representation, validated both visually and functionally through downstream classifier performance.
3. A systematic benchmark of 21 classifiers across eight model families, evaluated by held-out testing and group-aware five-fold cross-validation, with and without augmentation.
4. Detailed computational profiling (training time, inference latency, peak memory, model size) mapped to three practical deployment scenarios.

The remainder of this paper is organized as follows. Section “Experimental set-up and methods” describes the experimental setup, data acquisition, and the complete processing pipeline. Section “Results” presents the classification results, cross-validation analysis, and computational profiling. Section “Discussion” discusses the implications of the findings. Section “Conclusions” concludes with recommendations and future directions.

## Experimental set-up and methods

### Test facility and instrumentation

**Fig. 1:**
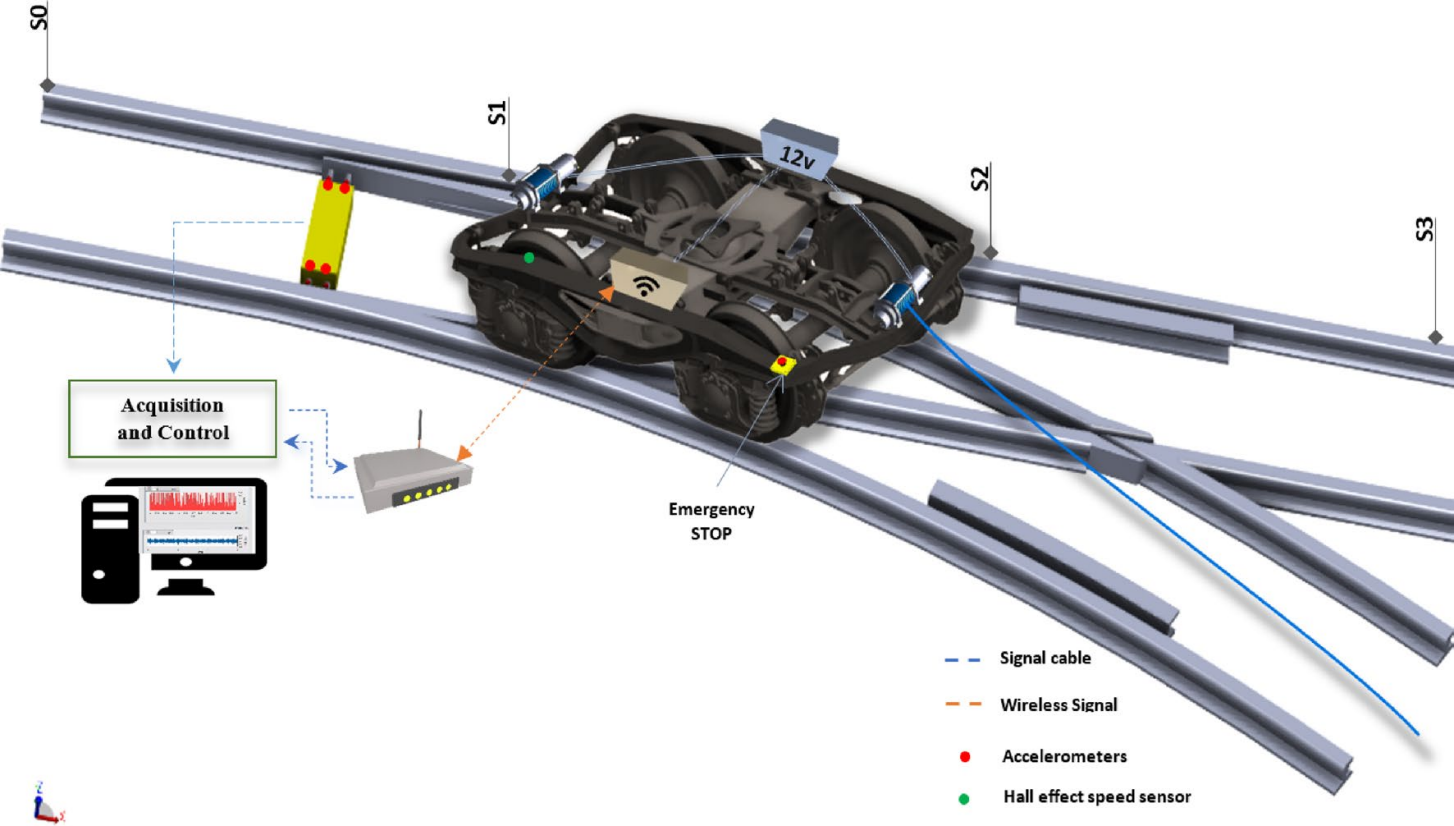
Schematic of the S&C test rig at the outdoor laboratory facility, showing the test bogie, point machine (yellow), accelerometer locations, and data acquisition system. Reproduced from Amin et al.^15^, *Transportation Engineering* **23**, 100414 (2026), under the Creative Commons Attribution (CC BY 4.0) license.

All experiments were conducted at a full-scale outdoor railway S&C test facility operated by Luleå University of Technology. This is a *controlled-laboratory* environment: defects are artificially induced and quantified, and excitation is generated under repeatable conditions. The facility comprises a complete S&C unit instrumented with accelerometers mounted within the point machine housing (Fig. 1). This placement provides physical protection for the sensors and enables integration as an optional feature within commercial point machines. Three factors support the feasibility of remote vibration detection from this location: (i) direct mechanical coupling between the sensors and switch blades via coupling rods; (ii) excellent vibration transmission through steel over considerable distances; and (iii) advanced signal processing and feature extraction techniques that can compensate for attenuation effects.

**Fig. 2:**
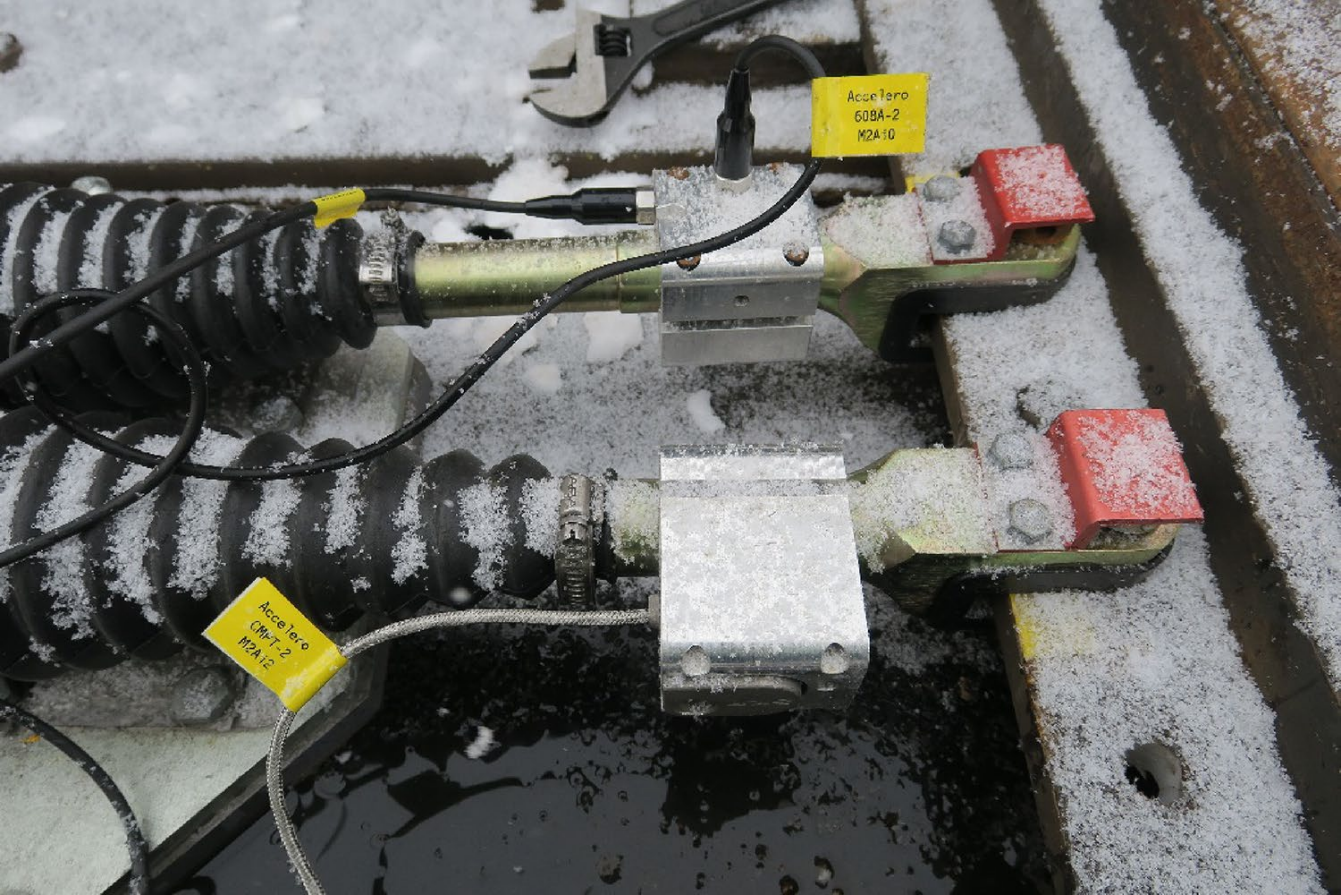
Accelerometers mounted on the coupling rods within the point machine housing. Aluminum sensor blocks are visible to the left of the yellow protective cover.

The sensor configuration comprises three accelerometers with 10 kHz bandwidth, covering longitudinal (*x*), lateral (*y*), and vertical (*z*) measurement directions. Table 1 summarizes the sensor specifications. Figure 2 shows the physical installation on the coupling rods.

**Table 1:** Accelerometer specifications and measurement directions.

Accelerometer ID	Module/Channel	Bandwidth	*x*	*y*	*z*
608A0	M1i1	10 kHz	$\checkmark$		
608A1	M1i2	10 kHz		$\checkmark$	
608A2	M2i0	10 kHz			$\checkmark$

### Test procedure and data acquisition

**Fig. 3:**
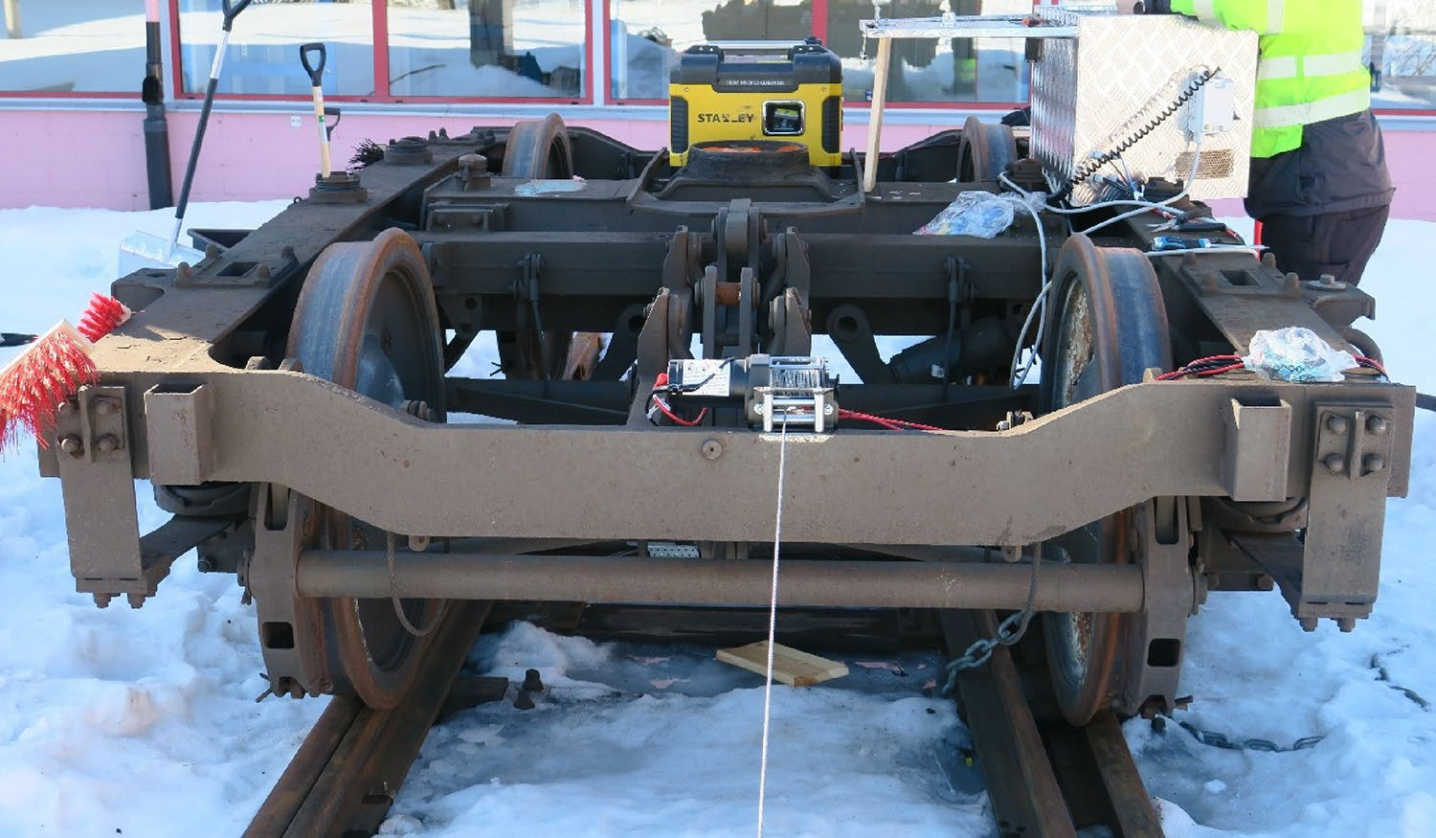
Test bogie with onboard petrol generator, data acquisition equipment, and winch cable.

A 6-tonne two-axle passenger train bogie (axle spacing 2500 mm) was used to generate vibration excitation (Fig. 3). The bogie was either towed bidirectionally by winches at approximately 0.05 m/s across three switch sections, or manually propelled at approximately 0.8 m/s over the entire switch length. Speed was measured using a hall-effect sensor mounted on the bogie. The winch system’s cable capacity necessitated dividing the test procedure into three sections: S0S1 (13.85 m, switch blade region), S1S2 (10.14 m, closure rail), and S2S3 (11.40 m, crossing section and exits). A National Instruments data acquisition system with wireless synchronization recorded all vibration signals.

**Fig. 4:**
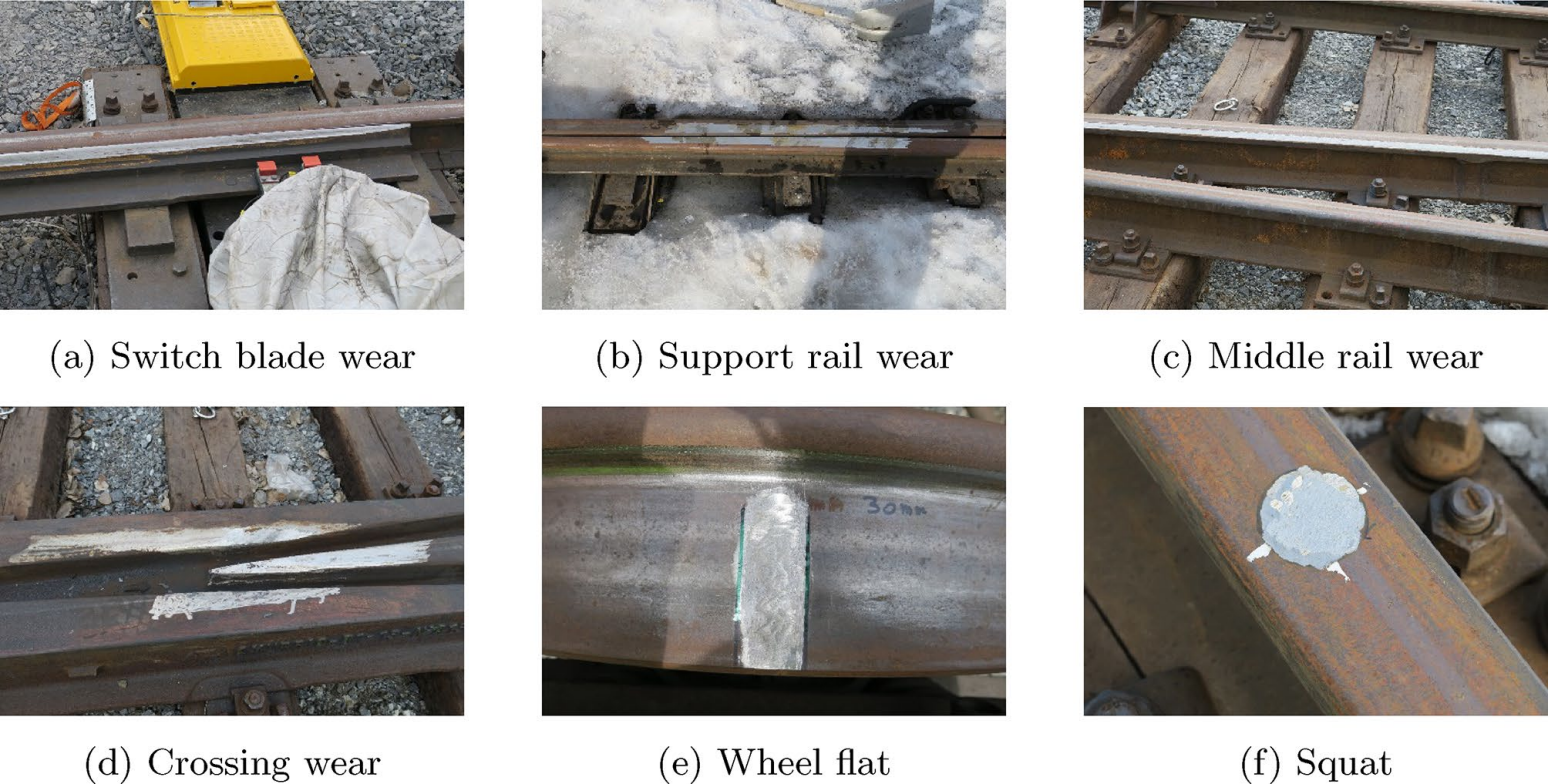
Examples of artificially induced defects at the test facility: (**a**) switch blade wear, (**b**) support rail wear, (**c**) middle rail wear, (**d**) crossing wear, (**e**) wheel flat, (**f**) squat. Reproduced from Amin et al.^15^, *Transportation Engineering* **23**, 100414 (2026), under the Creative Commons Attribution (CC BY 4.0) license.

Measurements were performed for four different wear and flaw severity levels on both the diverging and through tracks. Artificially induced wear and damage conditions were quantified using conventional mechanical measuring instruments. Figure 4 shows representative examples of the defect types investigated.

### Signal preprocessing

Raw vibration signals were recorded over complete bogie traversals, starting prior to movement initiation and ending after the stop block was reached. The signals were then preprocessed through a series of sequential steps. First, linear detrending was performed and the mean was adjusted to zero to remove static offsets and isolate dynamic variations in the signal. Next, a two-second Tukey window was applied symmetrically at the beginning and end of each time series to minimize edge effects. A second-order Butterworth bandpass filter with a frequency range of 1–8000 Hz was subsequently applied in both forward and backward directions to achieve zero-phase filtering, thereby reducing noise while preserving the relevant frequency content. After filtering, the tapered edges introduced by windowing were trimmed, retaining only the portion corresponding to the active bogie traversal between stop blocks. The signal, originally sampled at 51,200 Hz, was then downsampled by a factor of two to obtain a final sampling frequency of 25,600 Hz. Finally, the preprocessed data were divided into fixed one-second segments, and each segment was labeled according to predefined ground-truth categories: crossing, joint, normal, or squat.

**Fig. 5:**
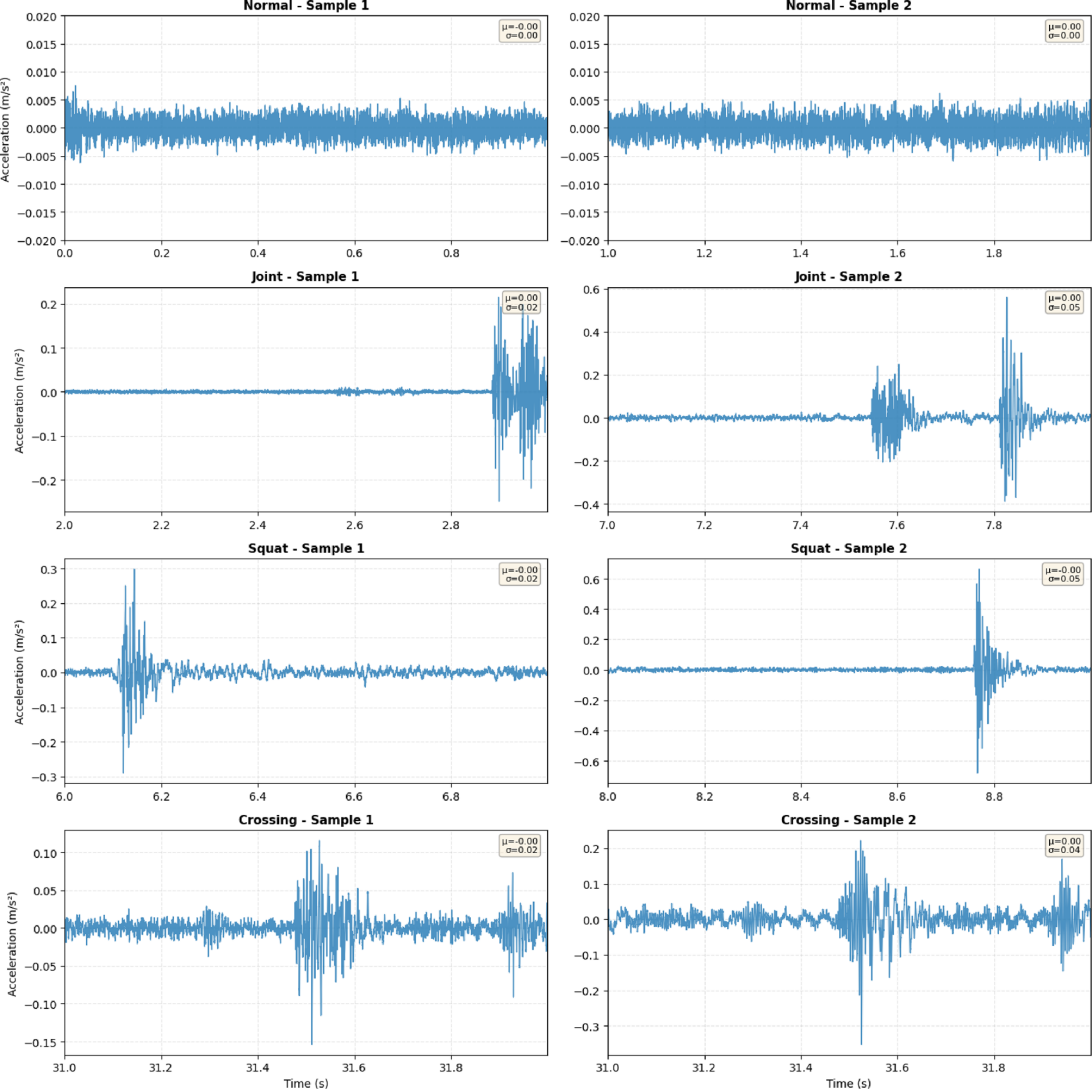
Representative vibration signal segments for the four event classes: normal, joint, squat, and crossing. Each row shows two samples per class.

Figure 5 shows representative vibration signal segments for each event class, illustrating the distinct dynamic characteristics: normal segments exhibit low-amplitude, relatively stationary vibrations; joint segments show impulsive, localized excitation; squat segments display broadband energy bursts; and crossing segments feature high-amplitude, complex waveforms.

### Feature engineering

Each one-second accelerometer segment is characterized by a 16-dimensional feature vector computed directly from the vibration (acceleration) signal of that segment. The three accelerometers (longitudinal, lateral, and vertical, Table 1) each produce their own segments; every segment is treated as an independent sample, and to prevent information leakage all segments originating from the same physical event are kept together during data partitioning (Section 2.5). The 16 features are grouped as follows.

#### Time-domain statistical descriptors (13 features)

Mean ($\mu$), standard deviation ($\sigma$), median, root mean square ($\text {RMS} = \sqrt{\frac{1}{N}\sum x_i^2}$), peak absolute value, peak-to-peak amplitude, skewness ($\gamma _1$), kurtosis ($\gamma _2$), energy ($E = \sum x_i^2$), crest factor (peak/RMS), first quartile ($Q_{25}$), third quartile ($Q_{75}$), and interquartile range ($\text {IQR} = Q_{75} - Q_{25}$).

#### Frequency-domain features (3 features)

Prior to spectral analysis, a Hanning window is applied to each segment to reduce spectral leakage, and the discrete Fourier transform *X*(*k*) is computed, where $k=1,\dots ,N/2$ indexes the non-negative frequency bins and the DC component ($k=0$) is excluded. The features are:

**Spectral centroid**: the amplitude-weighted mean frequency,

1 $$\begin{aligned} \text {SC} = \frac{\sum _k (f_k \cdot |X(k)|)}{\sum _k |X(k)|} \end{aligned}$$

where $f_k$ is the frequency of bin *k*, characterizing the spectral center of gravity.

**Spectral rolloff**: the frequency $f_r$ below which 85% of the total spectral energy is concentrated,

2 $$\begin{aligned} f_r = \min \left\{ f_k : \sum _{j \le k} |X(j)|^2 \ge 0.85 \cdot \sum _{j} |X(j)|^2 \right\} \end{aligned}$$

where *j* and *k* are frequency-bin indices. This energy-based definition provides a physically meaningful characterization of the signal’s frequency distribution.

**Dominant frequency**: the frequency component with the highest spectral magnitude,

3 $$\begin{aligned} f_{\text {dom}} = \arg \max _{f_k} |X(k)| \end{aligned}$$

### Evaluation pipeline

A central methodological contribution of this work is the enforcement of strict separation between training and test data at every pipeline stage. The pipeline proceeds as follows:

**Step 1 – Data loading and feature extraction**: Raw vibration segments are loaded and the 16-dimensional feature vector is extracted for every segment.

**Step 2 – Event-level stratified split**: The dataset is split into 80% training and 20% test subsets. Crucially, the split is performed at the level of *physical events*: each one-second event is recorded simultaneously by the three accelerometer axes, and all three resulting segments are assigned together to the same subset. A stratified, group-aware partitioning scheme (StratifiedGroupKFold) is used so that class proportions are preserved while no event is divided between training and test. This split is performed before any data-dependent transformation.

**Step 3 – Feature analysis (training data only)**: Mutual information (MI) between each feature and the class label is computed on the training set only and used to rank features by discriminative power. Given the already-low dimensionality (16 features), all features are retained for modelling; the MI ranking is used for interpretation (Section 3.2).

**Step 4 – Autoencoder training (training data only)**: The tabular autoencoder (Section 2.6) is trained exclusively on the training features.

**Step 5 – Class-balanced augmentation (training data only)**: Synthetic samples are generated only for the training set, expanding it from 639 to 1,055 samples.

**Step 6 – Feature standardization**: A StandardScaler (zero-mean, unit-variance) is fitted on the augmented training data and applied to both training and test sets, preventing test statistics from leaking into the scaling parameters.

**Step 7 – Model training and held-out evaluation**: Each classifier is trained on the augmented, standardized training set and evaluated on the unseen test set.

**Step 8 – Cross-validation**: Top-performing models undergo group-aware stratified five-fold cross-validation, with feature standardization applied independently within each fold, to provide robust generalization estimates with confidence intervals.

### Autoencoder-based data augmentation

**Fig. 6:**
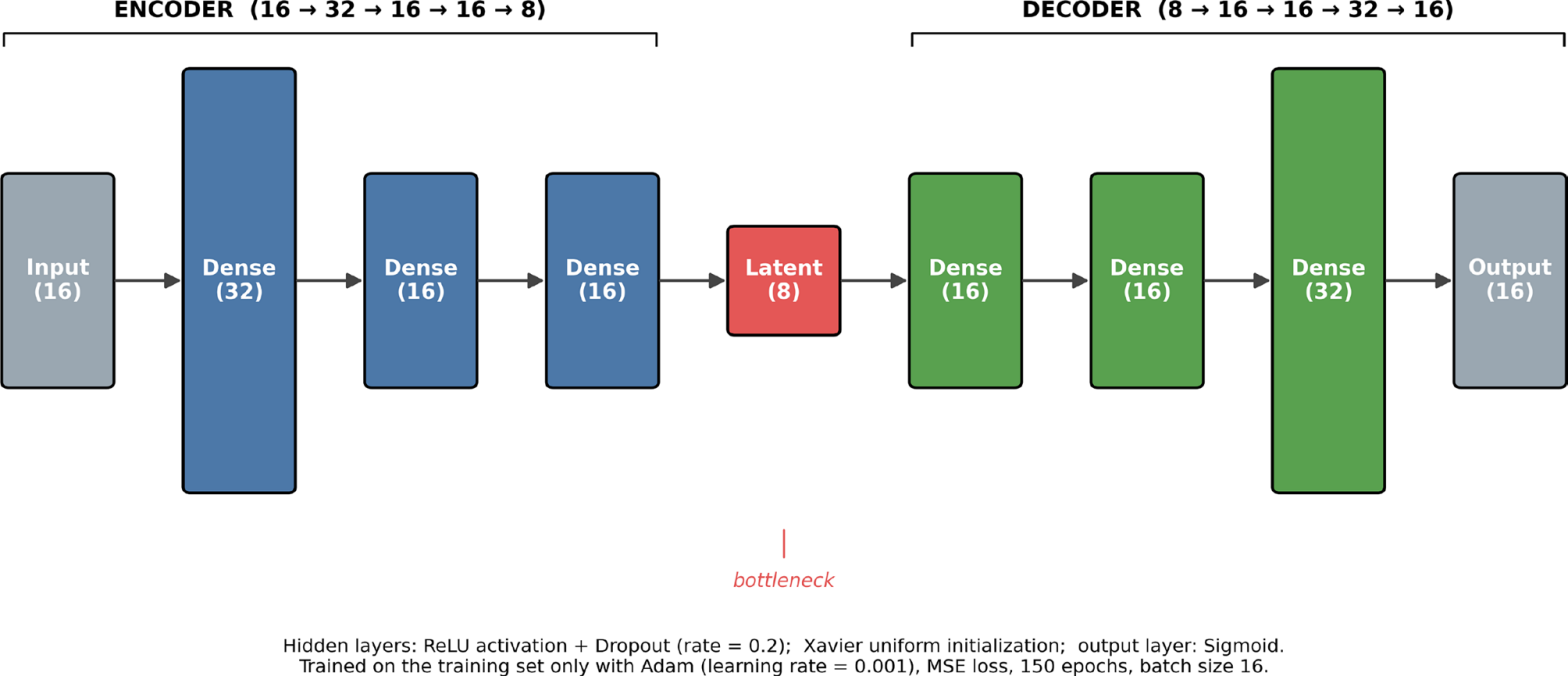
Architecture of the symmetric tabular autoencoder used for data augmentation. The encoder maps the 16-dimensional feature vector through dense layers ($32 \rightarrow 16 \rightarrow 16$) to an 8-dimensional latent bottleneck, and the decoder mirrors this structure. Hidden layers use ReLU activation with dropout (rate $=0.2$) and Xavier uniform initialization; the output layer uses a sigmoid activation. The autoencoder is trained on the training set only.

Given the limited dataset size and class imbalance, a symmetric tabular autoencoder is employed for data augmentation (Fig. 6). For the 16-dimensional input, the encoder compresses the input through hidden layers of width $32 \rightarrow 16 \rightarrow 16$ to an 8-dimensional latent representation, and the decoder mirrors this structure symmetrically. ReLU activations and dropout (rate $=0.2$) follow each hidden layer, and Xavier uniform initialization is used for weight stability. The latent dimension (8) is smaller than the input dimension (16), giving a conventional undercomplete autoencoder that learns a compact, denoised representation of the feature space.

The autoencoder is optimized using Adam (learning rate $=10^{-3}$) with MSE reconstruction loss over 150 epochs (batch size $=16$). Synthetic samples are generated by encoding training samples into the latent space, applying controlled Gaussian perturbation ($\sigma = 0.1$) to the latent vectors, and decoding back to feature space. The augmentation employs a class-balanced target ratio of 0.8 relative to the majority class: for each minority class, synthetic samples are generated until its count reaches $0.8 \times \max (n_c)$, specifically targeting under-represented classes. This expands the training set from 639 to 1,055 samples.

### Classification algorithms

Twenty-one classifiers spanning eight model families are evaluated to provide a comprehensive benchmark. Table 2 lists all models with their key hyperparameters. To ensure a uniform and reproducible comparison, hyperparameters were fixed *a priori* to widely used, literature-standard values rather than tuned individually per model. This deliberate choice avoids the optimistic bias that per-model tuning can introduce and ensures that observed performance differences reflect the algorithms themselves rather than unequal tuning effort. All values were set before any test-set evaluation, and the complete configuration is given in Table 2 so that the benchmark is fully reproducible.

**Table 2:** Classification algorithms and key hyperparameters.

Family	Algorithm	Key Hyperparameters
Tree-based	Random Forest	100 estimators, max depth 10, min split 5
	Extra Trees	100 estimators, max depth 10, min split 5
	Decision Tree	max depth 10, min split 5, min leaf 2
Boosting	Gradient Boosting	100 estimators, lr $=0.1$, max depth 6
	XGBoost	100 estimators, max depth 6, lr $=0.1$
	LightGBM	100 estimators, max depth 6, lr $=0.1$
	AdaBoost	100 estimators, lr $=1.0$
Linear	Logistic Regression	max_iter 1000, liblinear, one-vs-rest
	Ridge Classifier$^{*}$	$\alpha =1.0$, CalibratedCV, cv $=3$
	SGD Classifier$^{*}$	hinge loss, $\alpha =10^{-4}$, CalibratedCV
Instance-based	KNN ($k=5$)	uniform weights
	KNN ($k=10$)	distance weights
Neural network	MLP Small	(100), ReLU, 500 iter
	MLP Deep	(100, 50), ReLU, 500 iter
Probabilistic	Gaussian NB	default
	Bernoulli NB	$\alpha =1.0$
SVM	SVM Linear	$C=1.0$, linear kernel
	SVM RBF	$C=1.0$, $\gamma$ = scale
	SVM Poly	$C=1.0$, degree 3
Ensemble	Voting Classifier	soft voting (RF, XGBoost, Logistic Reg.)
	Bagging Classifier	50 estimators, Decision Tree base

$^{*}$Wrapped in CalibratedClassifierCV for probability estimates.

### Performance metrics and computational profiling

Classification performance is assessed using five metrics: accuracy, weighted precision, weighted recall, weighted F1-score, and ROC-AUC (one-vs-rest, weighted). For cross-validation, the mean and standard deviation of accuracy and F1-score across folds are reported.

Computational cost is profiled using Python’s tracemalloc and psutil libraries. The following metrics are recorded for each model: (i) wall-clock training time; (ii) wall-clock inference time; (iii) per-segment inference latency; (iv) peak memory consumption during training; (v) model serialization size (via pickle); and (vi) model parameter count. The inference latency is reported in milliseconds *per one-second segment*, i.e. the time to classify a single 16-dimensional feature vector (one segment), not the time per raw signal sample. All profiling was performed on a single workstation: Intel Core i7-10750H CPU (6 physical / 12 logical cores, 2.6 GHz), 32 GB RAM, Windows 11, Python 3.13, scikit-learn 1.8, NumPy 2.4, and PyTorch 2.11 (CPU only). Reported times are wall-clock measurements on this idle machine.

## Results

### Dataset characteristics

The dataset comprises 801 labeled one-second segments. These correspond to 267 distinct physical events, each recorded by the three accelerometer axes ($267 \times 3 = 801$). After the event-level stratified 80/20 split, the training set contains 639 segments and the test set 162 segments, with no event shared between them. Table 3 reports the exact class distribution before and after splitting, and after augmentation. The distribution is markedly imbalanced: the normal and squat classes dominate, while crossing is severely under-represented (only six segments in the test set). Augmentation rebalances the training set toward the majority class size.

**Table 3:** Class distribution before splitting, after the event-level 80/20 split, and after autoencoder augmentation of the training set.

Class	Total	Training	Test	Training (augmented)
Crossing	21	15	6	223
Joint	87	69	18	223
Normal	348	279	69	306
Squat	345	276	69	303
Total	801	639	162	1,055

### Feature importance analysis

**Fig. 7:**
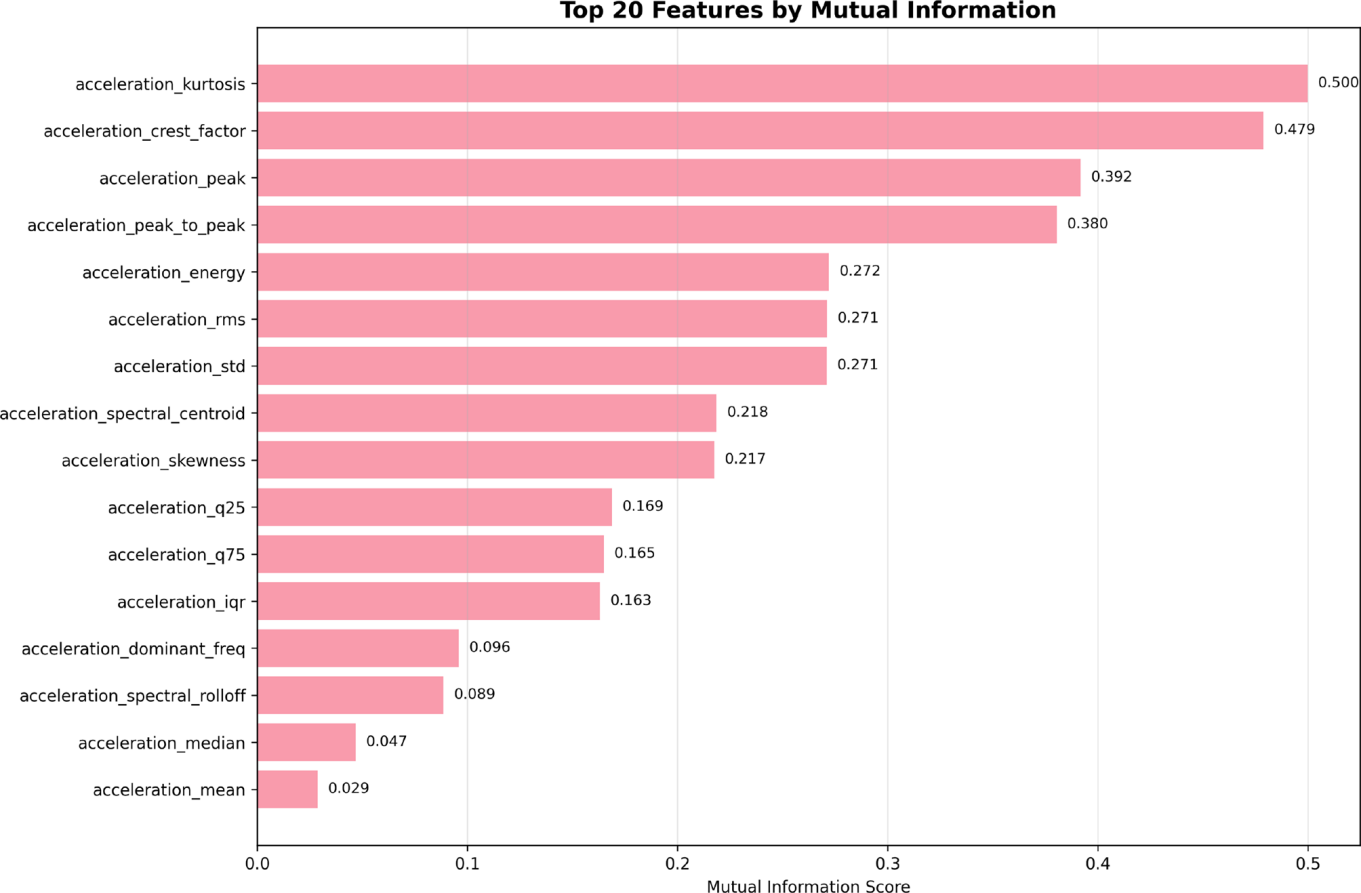
Features ranked by Mutual Information score on the training set. Amplitude shape descriptors (kurtosis, crest factor) achieve the highest discriminative power for event classification.

MI analysis of the 16 features (Fig. 7) reveals that amplitude shape descriptors provide the strongest discriminative power. Kurtosis achieves the highest MI score (0.50), followed by crest factor (0.48), peak (0.39), and peak-to-peak amplitude (0.38). These features capture the peakedness and impulsiveness of the vibration amplitude distribution, which are physically meaningful indicators of transient impact events at rail defects. Energy, RMS, and standard deviation form a second tier (MI $\approx 0.27$), while the spectral features and central-tendency statistics (median, mean) contribute least.

### Autoencoder training dynamics

**Fig. 8:**
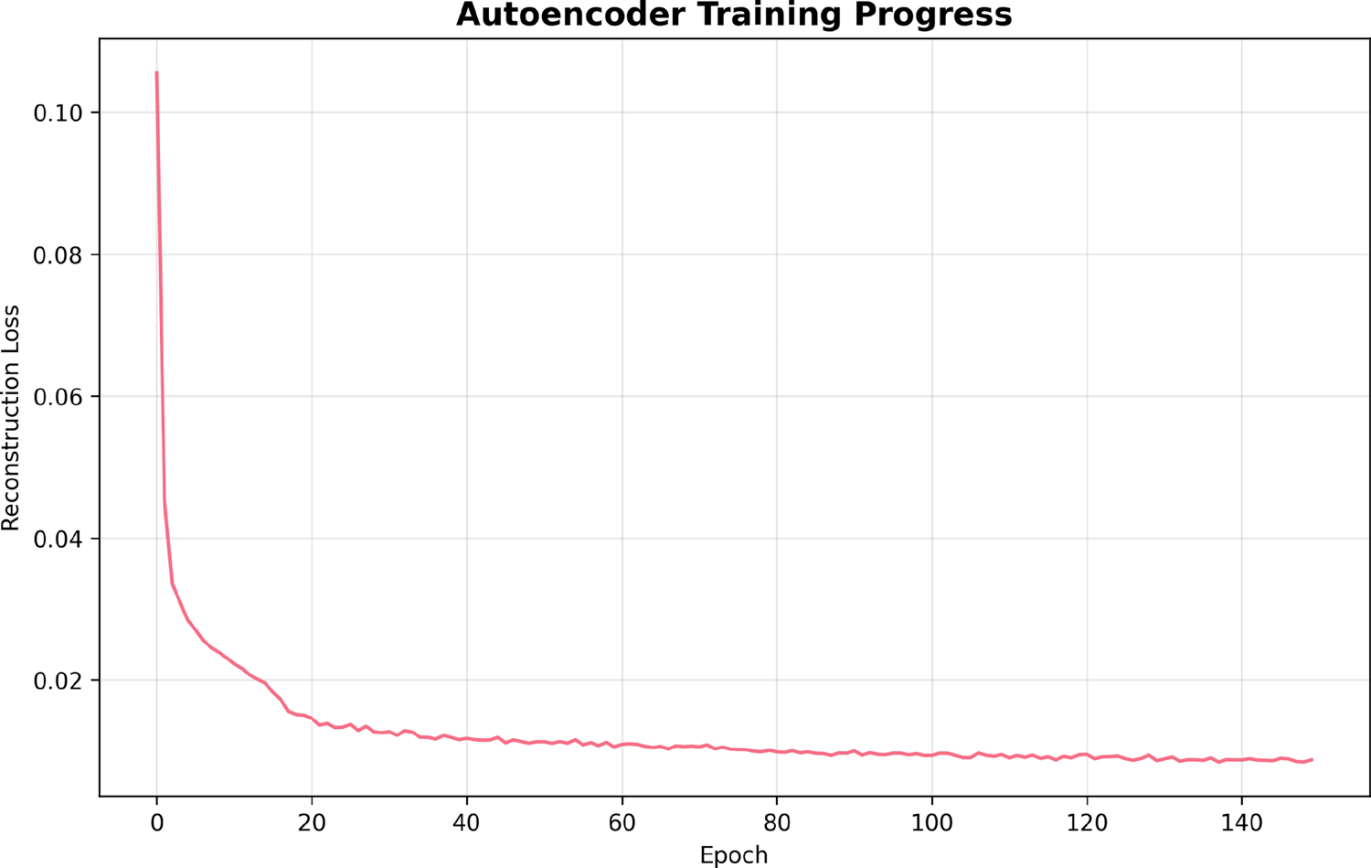
Autoencoder reconstruction loss over 150 training epochs, showing rapid convergence and a stable plateau.

The autoencoder reconstruction loss trajectory (Fig. 8) demonstrates effective convergence over 150 epochs. The loss descends rapidly during the first epochs and stabilizes near 0.009 thereafter. The smooth, monotonic decrease without oscillation indicates that the autoencoder learns a stable representation without overfitting to the limited training data.

### Data augmentation impact

**Fig. 9:**
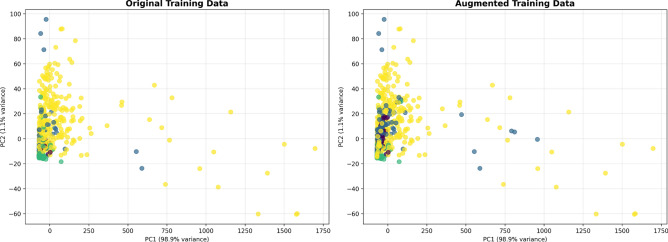
PCA visualization of training data before (left) and after (right) autoencoder-based augmentation. Synthetic samples expand minority-class clusters while preserving inter-class structure.

The class-balanced augmentation strategy expanded the training set from 639 to 1,055 samples. PCA visualization (Fig. 9) confirms that synthetic samples preserve the structural integrity of the data distribution: class clusters maintain their relative positions while minority-class clusters grow.

Beyond this visual check, the augmentation is validated *functionally*: every classifier is trained on the augmented (real + synthetic) data and evaluated on real held-out data only. Table 4 reports the resulting accuracy with and without augmentation. If the synthetic samples were not class-consistent, training on them would systematically degrade held-out accuracy; instead, the effect is small and model-dependent. Augmentation modestly improves several models, namely Gradient Boosting ($+1.9$ percentage points), Voting Classifier ($+1.9$), SGD ($+2.5$), MLP Deep ($+1.2$), and LightGBM ($+0.6$), and slightly reduces others, with the largest reductions for kernel- and distance-based methods (SVM Poly $-7.4$, SVM Linear $-6.8$, KNN $-5.6$). This pattern indicates that the synthetic samples preserve the global class structure (so ensemble and gradient-boosted models tolerate or benefit from them) but introduce mild local perturbations that affect algorithms relying on precise local geometry or kernel evaluations.

**Table 4:** Impact of data augmentation on held-out classification accuracy for representative models.

Model	Acc. (no aug.)	Acc. (aug.)	$\Delta$ Acc.
Gradient Boosting	0.778	0.796	$+0.019$
Voting Classifier	0.765	0.784	$+0.019$
MLP Deep	0.802	0.815	$+0.012$
LightGBM	0.790	0.796	$+0.006$
XGBoost	0.772	0.772	$\phantom {+}0.000$
Random Forest	0.784	0.765	$-0.019$
MLP Small	0.815	0.796	$-0.019$
Extra Trees	0.802	0.759	$-0.043$
KNN ($k=10$)	0.772	0.716	$-0.056$
SVM Linear	0.778	0.710	$-0.068$
SVM Poly	0.630	0.556	$-0.074$

### Comprehensive model performance evaluation

As reference points, a uniform-random classifier achieves 25.0% accuracy on this four-class problem, and a majority-class baseline (always predicting the most frequent class) achieves 42.6% on the test set. All 21 trained classifiers exceed these trivial baselines substantially (the strongest by nearly 40 percentage points over the majority-class baseline), confirming that the learned models capture genuine vibration-based discriminative information rather than class priors.

**Fig. 10:**
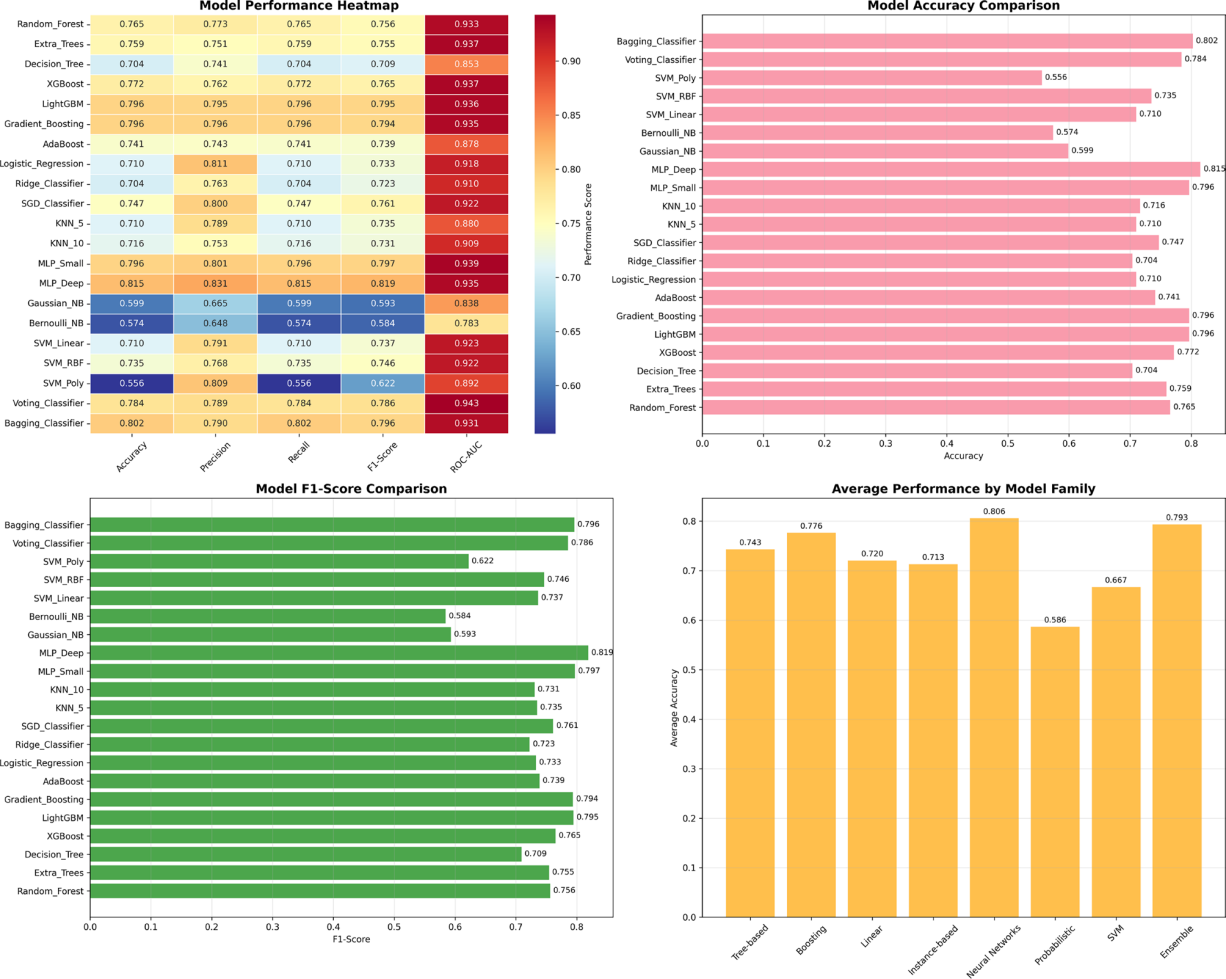
Comprehensive model performance: (**a**) performance heatmap across metrics, (**b**) accuracy ranking, (**c**) F1-score ranking, (**d**) average accuracy by model family.

**Fig. 11:**
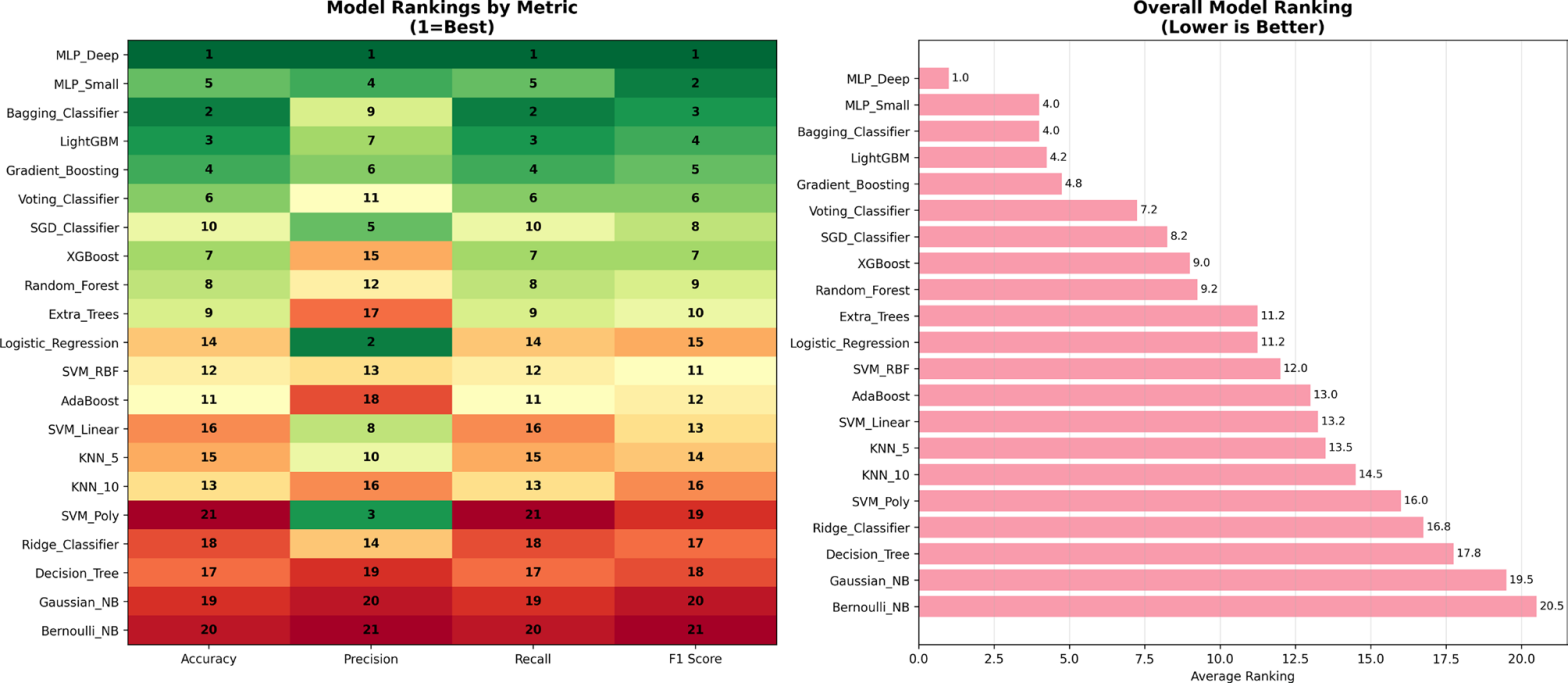
Model rankings by individual metric (left) and overall average ranking (right).

Table 5 presents the complete held-out test results for all 21 models with augmentation, and Table 6 the corresponding results without augmentation. Figure 10 provides the visual comparison and Fig. 11 the overall rankings.

**Table 5:** Classification performance of all 21 models (with augmentation), ranked by accuracy.

Rank	Model	Accuracy	Precision	Recall	F1-Score	ROC-AUC
1	MLP Deep	0.815	0.831	0.815	0.819	0.935
2	Bagging Classifier	0.802	0.790	0.802	0.796	0.931
3	LightGBM	0.796	0.795	0.796	0.795	0.936
4	Gradient Boosting	0.796	0.796	0.796	0.794	0.935
5	MLP Small	0.796	0.801	0.796	0.797	0.939
6	Voting Classifier	0.784	0.789	0.784	0.786	0.943
7	XGBoost	0.772	0.762	0.772	0.765	0.937
8	Random Forest	0.765	0.773	0.765	0.756	0.933
9	Extra Trees	0.759	0.751	0.759	0.755	0.937
10	SGD Classifier	0.747	0.800	0.747	0.761	0.922
11	AdaBoost	0.741	0.743	0.741	0.739	0.878
12	SVM RBF	0.735	0.768	0.735	0.746	0.922
13	KNN ($k=10$)	0.716	0.753	0.716	0.731	0.909
14	Logistic Regression	0.710	0.811	0.710	0.733	0.918
15	KNN ($k=5$)	0.710	0.789	0.710	0.735	0.880
16	SVM Linear	0.710	0.791	0.710	0.737	0.923
17	Decision Tree	0.704	0.741	0.704	0.709	0.853
18	Ridge Classifier	0.704	0.763	0.704	0.723	0.910
19	Gaussian NB	0.599	0.665	0.599	0.593	0.838
20	Bernoulli NB	0.574	0.648	0.574	0.584	0.783
21	SVM Poly	0.556	0.809	0.556	0.622	0.892

**Table 6:** Classification performance of all 21 models (without augmentation), ranked by accuracy.

Rank	Model	Accuracy	Precision	Recall	F1-Score	ROC-AUC
1	MLP Small	0.815	0.813	0.815	0.810	0.945
2	Bagging Classifier	0.815	0.816	0.815	0.812	0.933
3	Extra Trees	0.802	0.763	0.802	0.779	0.944
4	MLP Deep	0.802	0.820	0.802	0.803	0.946
5	AdaBoost	0.796	0.762	0.796	0.777	0.901
6	LightGBM	0.790	0.796	0.790	0.789	0.935
7	Random Forest	0.784	0.787	0.784	0.774	0.935
8	Gradient Boosting	0.778	0.769	0.778	0.772	0.930
9	SVM Linear	0.778	0.743	0.778	0.757	0.928
10	SVM RBF	0.778	0.733	0.778	0.738	0.932
11	XGBoost	0.772	0.776	0.772	0.766	0.939
12	KNN ($k=10$)	0.772	0.730	0.772	0.748	0.926
13	KNN ($k=5$)	0.765	0.758	0.765	0.761	0.904
14	Voting Classifier	0.765	0.738	0.765	0.749	0.942
15	Ridge Classifier	0.747	0.635	0.747	0.685	0.918
16	Decision Tree	0.728	0.726	0.728	0.721	0.843
17	Logistic Regression	0.728	0.629	0.728	0.671	0.921
18	SGD Classifier	0.722	0.618	0.722	0.660	0.920
19	Gaussian NB	0.642	0.694	0.642	0.615	0.880
20	SVM Poly	0.630	0.575	0.630	0.568	0.877
21	Bernoulli NB	0.617	0.643	0.617	0.597	0.813

Several key findings emerge. First, the strongest models (the deep MLP, the Bagging ensemble, LightGBM, Gradient Boosting, and the small MLP) cluster closely around 80–82% held-out accuracy, with ROC-AUC values near 0.93–0.94. Neural-network and ensemble methods are comparably effective on this dataset; no single family dominates the held-out ranking. This contrasts with the pattern frequently reported for tabular data, where tree-based models tend to outperform neural networks^22^: with the present compact 16-feature representation, the two families perform comparably. Second, all 21 models converge successfully, confirming the critical role of feature standardization: in preliminary experiments without standardization, MLP models collapsed far below the random-chance level of 25%. Third, probabilistic classifiers (Gaussian and Bernoulli Naive Bayes) and the polynomial-kernel SVM are the weakest performers, consistent with their stronger distributional assumptions.

### Cross-validation results

**Fig. 12:**
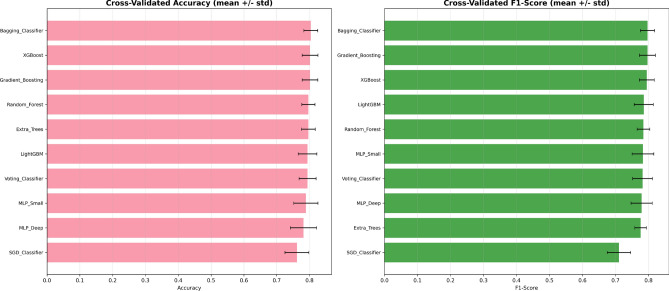
Group-aware five-fold cross-validation results: mean accuracy (left) and F1-score (right) with error bars indicating standard deviation.

Group-aware stratified five-fold cross-validation was conducted for the top 10 models, with feature standardization applied independently within each fold and with events kept intact across folds. Table 7 presents the results, and Fig. 12 the corresponding visualization.

**Table 7:** Group-aware five-fold cross-validation results (top 10 models).

Model	Mean accuracy	$\sigma$ accuracy	Mean F1	$\sigma$ F1
Bagging Classifier	0.804	±0.021	0.797	±0.021
XGBoost	0.802	±0.024	0.795	±0.022
Gradient Boosting	0.802	±0.024	0.797	±0.024
Random Forest	0.797	±0.020	0.785	±0.019
Extra Trees	0.797	±0.021	0.776	±0.018
LightGBM	0.794	±0.028	0.786	±0.029
Voting Classifier	0.794	±0.026	0.782	±0.030
MLP Small	0.789	±0.037	0.783	±0.033
MLP Deep	0.782	±0.040	0.779	±0.032
SGD Classifier	0.762	±0.036	0.710	±0.035

Cross-validation refines the picture from the single held-out split. The ensemble methods, namely Bagging ($80.4\%\pm 2.1\%$), XGBoost ($80.2\%\pm 2.4\%$), and Gradient Boosting ($80.2\%\pm 2.4\%$), achieve the highest mean accuracy and, importantly, the lowest variance. The two neural networks, although competitive on the single held-out split, show larger fold-to-fold variance ($\pm 3.7$ to $\pm 4.0$ percentage points). For deployment decisions, where stability across data partitions matters, the ensemble methods are therefore the most reliable choice. The close agreement between cross-validation means and held-out accuracy (within a few percentage points) supports the validity of the evaluation methodology.

### Confusion matrix analysis

**Fig. 13:**
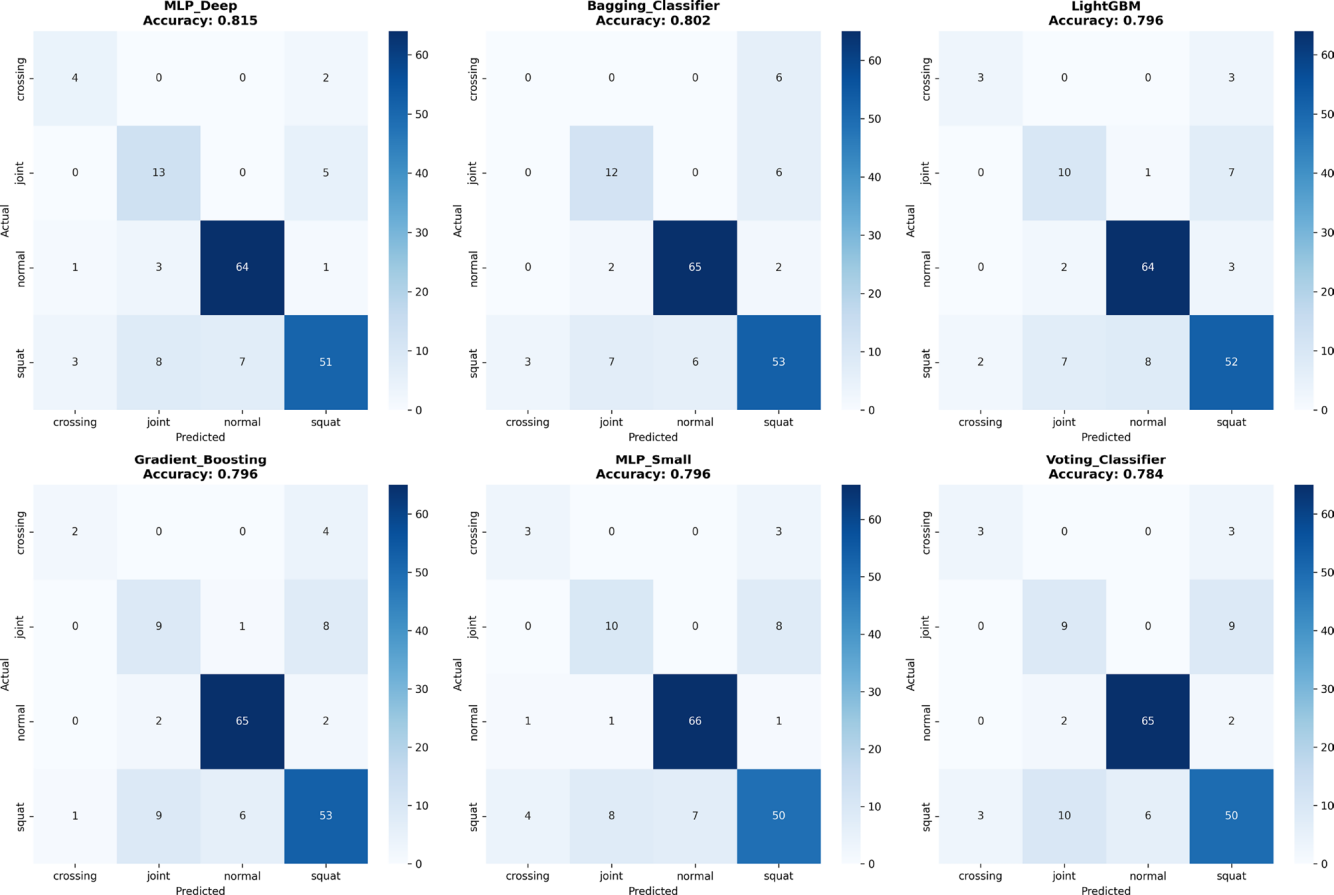
Confusion matrices for the top six models (with augmentation). Darker diagonal elements indicate higher classification accuracy.

Figure 13 presents confusion matrices for the top six models. Consistent class-specific patterns emerge:

**Normal class**: highest recall (93–96%), benefiting from its large representation and distinctive low-amplitude signature.

**Squat**: moderate-to-strong detection (72–77% recall), with occasional confusion with normal and joint.

**Joint**: moderate performance (50–72% recall), most often confused with squat, suggesting partial spectral overlap between the two.

**Crossing**: the most challenging class. With only six held-out samples, recall ranges from 0% to 67% across the top models, and a single misclassification shifts recall by roughly 17 percentage points. Exact (Clopper–Pearson) 95% confidence intervals make this uncertainty explicit: for example, a model classifying 3 of 6 crossing samples correctly (recall $=50\%$) has a 95% CI of $[12\%,\,88\%]$, and one classifying 4 of 6 correctly (recall $=67\%$) has a CI of $[22\%,\,96\%]$. Per-class metrics for the crossing class are therefore not statistically reliable and are reported for completeness only. Expanding this class is a priority for future work.

### Computational resource profiling

**Fig. 14:**
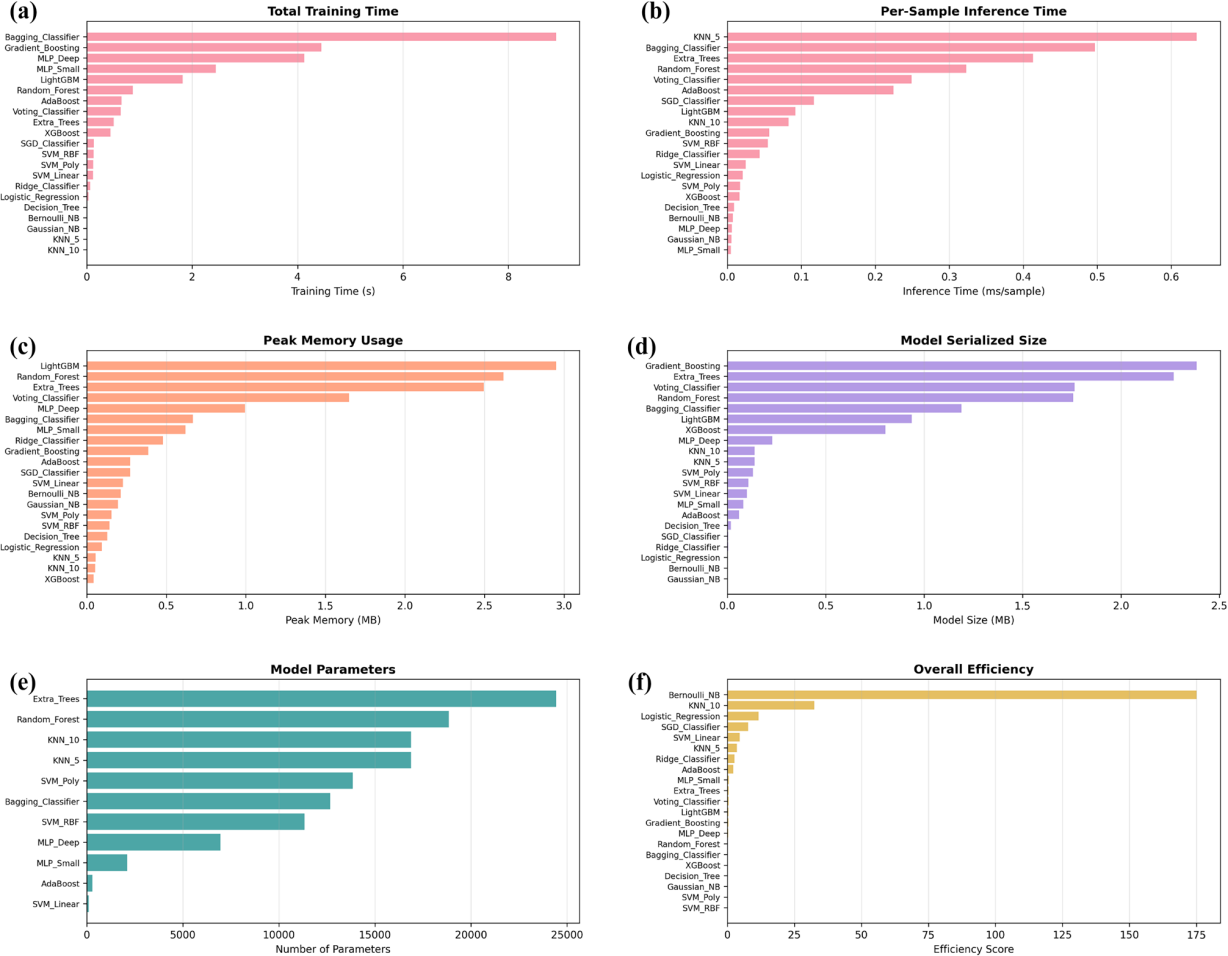
Computational resource profiling: (**a**) training time, (**b**) per-segment inference time, (**c**) peak memory usage, (**d**) model serialized size, (**e**) parameter count, (**f**) overall efficiency score.

Table 8 presents the computational resource requirements, and Fig. 14 the detailed profiling visualizations.

**Table 8:** Computational resource profiling for selected models (with augmentation). Inference latency is reported per one-second segment.

Model	Train (s)	Infer. (ms/segment)	Peak Mem. (MB)	Size (MB)	Accuracy
MLP Deep	4.13	0.006	0.99	0.228	0.815
Bagging Classifier	8.90	0.497	0.67	1.189	0.802
LightGBM	1.82	0.092	2.95	0.937	0.796
Gradient Boosting	4.45	0.057	0.39	2.385	0.796
MLP Small	2.45	0.005	0.62	0.080	0.796
XGBoost	0.45	0.016	0.04	0.803	0.772
Random Forest	0.87	0.323	2.62	1.757	0.765
Decision Tree	0.012	0.009	0.13	0.018	0.704
Logistic Regression	0.036	0.021	0.09	0.002	0.710
KNN ($k=5$)	0.001	0.635	0.06	0.138	0.710
Gaussian NB	0.002	0.005	0.20	0.002	0.599

Training time spans four orders of magnitude, from 0.001 s (KNN) to 8.9 s (Bagging). Inference latency, measured per one-second segment, ranges from 0.005 ms (MLP Small) to 0.635 ms (KNN, $k=5$); even the slowest model classifies a segment in well under a millisecond, far faster than the one-second acquisition window. Peak memory consumption stays below 3 MB for every model, and serialized model sizes range from 0.002 MB to 2.4 MB, all comfortably within the constraints of modern embedded systems.

### Deployment scenario analysis

Based on the combined performance–efficiency analysis, three deployment scenarios are identified:

**Scenario 1: Maximum reliability (safety-critical applications).** The ensemble methods (Bagging, XGBoost, and Gradient Boosting) are recommended, as they achieve the highest cross-validated accuracy ($\approx 80\%$) with the lowest variance ($\pm 2$–2.4 percentage points). Their stability across data partitions is the decisive property for safety-critical use.

**Scenario 2: Real-time edge computing.** XGBoost offers an excellent balance of accuracy (77%), very low inference latency (0.016 ms/segment), and minimal peak memory (0.04 MB). For the lowest possible latency, the MLP models classify a segment in 0.005–0.006 ms.

**Scenario 3: Memory-constrained embedded systems.** Logistic Regression offers the smallest footprint (0.002 MB model, 0.09 MB peak memory); the Decision Tree provides a similarly compact, fully interpretable alternative.

## Discussion

### Methodological implications of leak-free evaluation

The enforcement of strict pipeline ordering, together with event-level partitioning, is a critical methodological safeguard. Because each physical event is observed by three accelerometer axes, a naive random split could place correlated measurements of the same event in both the training and the test set. Partitioning at the event level removes this possibility, so that the held-out set is genuinely independent. Combined with applying all data-dependent transformations (feature analysis, autoencoder training, augmentation, standardization) to the training data only, this ensures that reported metrics reflect true out-of-sample generalization. We recommend that future studies in this domain adopt similar discipline and report their partitioning scheme explicitly.

### Critical role of feature standardization

The impact of feature standardization deserves attention. Vibration features span very different numerical ranges; without normalization, gradient-based optimization in MLPs and distance or kernel computations in KNN and SVMs are dominated by high-magnitude features. After standardization, all 21 models converge and produce meaningful predictions. This underscores that standardization is not merely a recommended practice but a requirement for any pipeline involving scale-sensitive algorithms. The robustness of tree-based methods to unstandardized features may explain why this issue is under-reported in studies focusing exclusively on tree-based classifiers.

### When should augmentation be used?

The model-dependent impact of augmentation (Table 4) supports a practical decision rule. Augmentation helped or left unchanged the gradient-boosted and meta-ensemble models (Gradient Boosting, LightGBM, Voting) and the deeper neural network, but reduced the accuracy of distance-based (KNN) and kernel-based (SVM) models. The underlying reason is that autoencoder-generated samples preserve the *global* class distribution but introduce mild *local* perturbations. We therefore recommend the following heuristic: apply autoencoder augmentation when the target classifier aggregates many weak partitions of the feature space (boosting, bagging, voting) or learns smooth global decision functions, but evaluate it cautiously, against a non-augmented baseline, for classifiers that depend on precise local geometry (KNN) or kernel evaluations (SVM). In all cases, both pipelines should be compared on held-out data before deployment.

### Cross-validation as a reliability metric

The cross-validation results (Table 7) refine the held-out ranking in an operationally important way. While the deep MLP attains the single highest held-out accuracy, it also shows the largest fold-to-fold variance; the ensemble methods are marginally lower on average but substantially more stable. For deployment decisions, cross-validation performance, which integrates information across multiple partitions, should be the primary selection criterion over any single split. On this basis, ensemble methods are the recommended default for this application.

### Comparison with existing approaches

Direct comparison with existing studies is constrained by differences in datasets, preprocessing, and evaluation protocols. Chen et al.^17^ note that tree-based classifiers typically achieve 85–92% on railway fault datasets that are considerably larger than ours. Our leak-free results ($\approx 80$–$82\%$) are lower, which is expected: the present dataset is small, the evaluation is strictly leak-free and event-partitioned, and no per-model tuning was applied. Compared to deep-learning approaches for rail defect detection^14^, our tabular feature-based framework offers far lower computational requirements while remaining suitable for edge deployment. Multi-modal approaches^23^ may improve accuracy further at the cost of system complexity.

### Limitations

Several limitations should be emphasized. First, the dataset is collected from a single S&C test facility with artificially induced defects under controlled conditions. The results should therefore be regarded as a *controlled-laboratory benchmark*; performance on operational railway infrastructure (with naturally occurring degradation, variable train speeds, environmental noise, and traffic-dependent loading) remains to be validated, and any claim of operational readiness would be premature until such field validation is carried out. Second, the dataset is small (267 physical events), and the crossing class is severely under-represented (six held-out segments), so its per-class metrics are not statistically reliable, as quantified by the wide confidence intervals in Section 3.7. Third, the persistent confusion between joint and squat classes suggests that additional features capturing temporal localization or higher-order spectral characteristics may be needed.

## Conclusions

This study presented a rigorously structured, leak-free machine-learning framework for vibration-based event classification in railway switches and crossings, evaluated on a controlled-laboratory dataset from a full-scale outdoor test facility. The main findings are:

- **Leak-free, event-partitioned evaluation.** Enforcing the ordering split $\rightarrow$ select $\rightarrow$ augment $\rightarrow$ standardize $\rightarrow$ train $\rightarrow$ evaluate, and partitioning at the level of physical events, yields performance estimates that reflect genuine generalization.
- **Accuracy.** The strongest models reach 81.5% held-out accuracy (ROC-AUC $\approx 0.94$); group-aware cross-validation gives $\approx 80\%$ for the leading models, all far above the 25% random and 42.6% majority-class baselines.
- **Model choice.** Ensemble methods (Bagging, XGBoost, Gradient Boosting) provide the most stable cross-validated performance and are recommended as the default; neural networks are competitive but more variable.
- **Standardization** is essential: without it, scale-sensitive models fail entirely.
- **Augmentation** has a small, model-dependent effect: beneficial for ensemble and gradient-boosted models, detrimental for distance- and kernel-based models.
- **Deployment.** All models satisfy embedded-system constraints, with sub-millisecond per-segment inference and model sizes below 2.4 MB.

**Limitations.** The study is a controlled-laboratory benchmark with artificially induced defects; the dataset is small; and the crossing class is too under-represented for reliable per-class evaluation.

**Future work.** Priorities are: (i) expanding the dataset, particularly the under-represented classes; (ii) validating the models on operational railway infrastructure; (iii) investigating transfer learning for cross-site generalization; and (iv) incorporating temporal sequence modelling.

## Data Availability

The extracted-feature dataset analysed during the current study is openly available in the Zenodo repository: https://doi.org/10.5281/zenodo.20715840 (DOI 10.5281/zenodo.20715840). The raw segmented vibration recordings are large in volume and are available from the corresponding author on reasonable request.

## Code availability

The complete analysis code (feature extraction, leak-free pipeline, autoencoder augmentation, and benchmarking) together with the extracted-feature dataset required to reproduce every result reported in this study is openly available in the Zenodo repository: https://doi.org/10.5281/zenodo.20715840 (DOI 10.5281/zenodo.20715840).

## References

[1] Gonzalo, A. P., Horridge, R., Steele, H., Stewart, E. & Entezami, M. Review of data analytics for condition monitoring of railway track geometry. *IEEE Trans. Intell. Transp. Syst.* **23**, 22737–22754 (2022).

[2] Sedghi, M., Kauppila, O., Bergquist, B., Vanhatalo, E. & Kulahci, M. A taxonomy of railway track maintenance planning and scheduling: a review and research trends. *Reliab. Eng. Syst. Saf.* **215**, 107827 (2021).

[3] dos Santos, J. M. O., Bressi, S. & Losa, M. Optimization of maintenance strategies for railway track-bed considering probabilistic degradation models and different reliability levels. *Reliab. Eng. Syst. Saf.* **207**, 107359 (2021).

[4] Tang, R. et al. A literature review of Artificial Intelligence applications in railway systems. *Transp. Res. Part C* **140**, 103679 (2022).

[5] Consilvio, A., Di Febbraro, A., Meo, R. & Sacco, N. Risk-based optimal scheduling of maintenance activities in a railway network. *EURO J. Transp. Logist.* **8**, 435–465 (2019).

[6] Micic, M., Brajovic, L., Lazarevic, L. & Popovic, Z. Inspection of RCF rail defects - review of NDT methods. *Mech. Syst. Signal Process.* **182**, 109568 (2023).

[7] Liang, B., Iwnicki, S., Ball, A. & Young, A. E. Adaptive noise cancelling and time-frequency techniques for rail surface defect detection. *Mech. Syst. Signal Process.* **54**, 41–51 (2015).

[8] Hodge, V. J., O’Keefe, S., Weeks, M. & Moulds, A. Wireless sensor networks for condition monitoring in the railway industry: a survey. *IEEE Trans. Intell. Transp. Syst.* **16**, 1088–1106 (2015).

[9] Du, C., Dutta, S., Kurup, P., Yu, T. & Wang, X. A review of railway infrastructure monitoring using fiber optic sensors. *Sens. Actuators A: Phys.* **303**, 111728 (2020).

[10] Kasraei, A. et al. Climate change impacts assessment on railway infrastructure in urban environments. *Sustain. Cities Soc.* **101**, 105084 (2024).

[11] Mosleh, A., Meixedo, A., Ribeiro, D., Montenegro, P. & Calçada, R. Automatic clustering-based approach for train wheels condition monitoring. *Int. J. Rail Transp.* **11**, 639–664 (2023).

[12] Ghiasi, R., Khan, M. A., Sorrentino, D., Diaine, C. & Malekjafarian, A. An unsupervised anomaly detection framework for onboard monitoring of railway track geometrical defects using one-class support vector machine. *Eng. Appl. Artif. Intell.* **133**, 108167 (2024).

[13] Siddiqui, H. U. R. et al. IoT based railway track faults detection and localization using acoustic analysis. *IEEE Access* **10**, 106520–106533 (2022).

[14] De Donato, L. et al. A survey on audio-video based defect detection through deep learning in railway maintenance. *IEEE Access* **10**, 65376–65400 (2022).

[15] Amin, M. A., Najeh, T., Sridharan, N. V., Ghoul, A. & Karim, R. Enhancing railway infrastructure monitoring with AI: a machine learning approach for event detection. *Transp. Eng.* **23**, 100414 (2026).

[16] Amin, M. A. et al. AI-enhanced rail infrastructure monitoring using multi-sensor vibration data: a case study in Sweden. In *International Congress and Workshop on Industrial AI and eMaintenance 2025, Lecture Notes in Mechanical Engineering* 811–824 (Springer, 2026).

[17] Chen, H., Jiang, B., Ding, S. X. & Huang, B. Data-driven fault diagnosis for traction systems in high-speed trains: a survey, challenges, and perspectives. *IEEE Trans. Intell. Transp. Syst.* **23**, 1700–1716 (2022).

[18] Kapoor, S. & Narayanan, A. Leakage and the reproducibility crisis in machine-learning-based science. *Patterns* **4**, 100804 (2023). 37720327 10.1016/j.patter.2023.100804 PMC10499856

[19] Fernandez-Bobadilla, H. A., Bouchikhi, Y. & Martin, U. GAN-based data augmentation of time series for fault diagnosis in railway track. *Railway Eng. Sci.* (2025).

[20] Gbadamosi, A.-Q. et al. IoT for predictive assets monitoring and maintenance: An implementation strategy for the UK rail industry. *Autom. Constr.* **122**, 103486 (2021).

[21] Cheng, J. C. P., Chen, W., Chen, K. & Wang, Q. Data-driven predictive maintenance planning framework for MEP components based on BIM and IoT using machine learning algorithms. *Autom. Constr.* **112**, 103087 (2020).

[22] Grinsztajn, L., Oyallon, E. & Varoquaux, G. Why do tree-based models still outperform deep learning on typical tabular data?. *Adv. Neural Inf. Process. Syst.* **35**, 507–520 (2022).

[23] Lederman, G. et al. A data fusion approach for track monitoring from multiple in-service trains. *Mech. Syst. Signal Process.* **95**, 363–379 (2017).

